# Synergistic remediation of aqueous Cd(ii) by sewage sludge biochar *via* P/Fe co-impregnation

**DOI:** 10.1039/d5ra09939k

**Published:** 2026-02-23

**Authors:** Yunping Ji, Yarong Zhao, Qingfeng Lv, Fei Gao

**Affiliations:** a School of Civil Engineering and Mechanics, Lanzhou University Lanzhou 730000 China shaopei13698@163.com; b China Railway First Survey and Design Institute Group Co., Ltd Lanzhou 730000 China; c Zhejiang Huancheng Environmental Protection Technology Co., Ltd Hangzhou 310012 China; d China Railway 21st Bureau Group Co., Ltd Lanzhou 730070 China

## Abstract

Converting municipal sewage sludge into high-efficiency adsorbents represents a sustainable strategy for cadmium [Cd(ii)] remediation in acid mine drainage (AMD) and for solid-waste valorization. A novel phosphorus/iron co-modified sludge biochar (P–Fe@SBC) was synthesized *via* a combined FeCl_3_–KH_2_PO_4_ impregnation and pyrolysis route. Modification improved the microstructure. The specific surface area of P–Fe@SBC increased to 137.915 m^2^ g^−1^, 7.4 times that of pristine biochar. Adsorption tests demonstrated outstanding Cd(ii) removal. Adsorption conformed to the Langmuir isotherm model and the pseudo-second-order kinetic model. The maximum removal capacity reached 328.95 mg g^−1^, markedly exceeding that of singly modified biochars and pristine biochar. High selectivity was observed under complex ionic matrices (K^+^, Ca^2+^, Mg^2+^). Approximately 90% of the removal amount remained after five adsorption–desorption cycle, indicating high stability and strong regeneration potential. Mechanistic analyses indicated a synergistic removal network involving electrostatic attraction, chemical precipitation, inner-sphere surface complexation, cation–π interaction, and ion exchange. P–Fe@SBC represented a promising waste-derived material for “waste-to-treat-waste” remediation.

## Abstract 2

Converting municipal sewage sludge into high-efficiency adsorbents represents a sustainable strategy for cadmium [Cd(ii)] remediation in acid mine drainage (AMD) and for solid-waste valorization.

## Graphical abstract

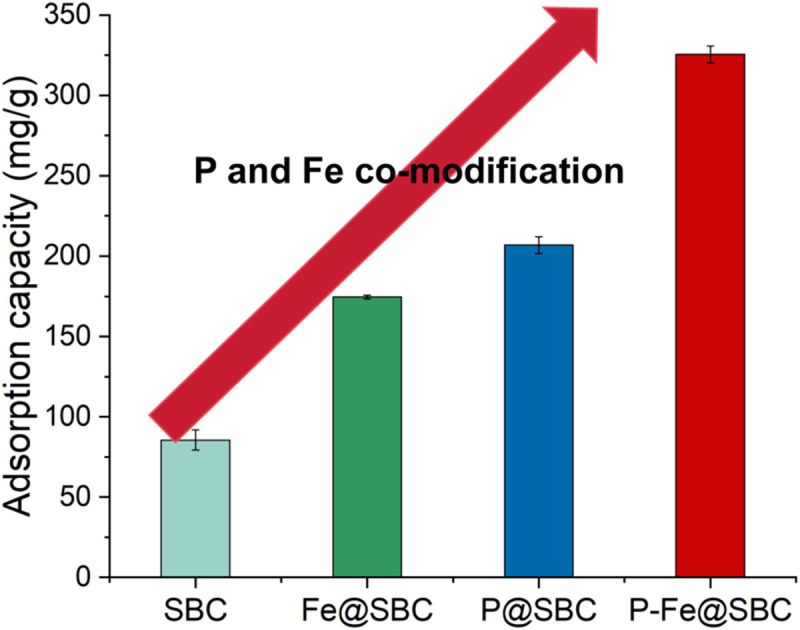

## 1 Introduction

With the exploitation of mineral resources, large volumes of acid mine drainage (AMD) with pH values of 3–6 are discharged into natural environments.^1,2^ AMD originates from mine water during extraction, beneficiation effluents, and leachates from tailings and waste-rock dumps.^3^ Such wastewater exhibits high chemical complexity and typically contains multiple heavy metals, including Pb, Cu, Zn, and Cd.^2^ Once released, AMD undergoes transport and accumulation in environmental media, posing sustained ecological risks. Cadmium [Cd(ii)] is a ubiquitous contaminant in AMD, characterized by high mobility, strong toxicity, wide contamination footprints, non-degradability, and pronounced bioaccumulation potential.^4,5^ Once released into the environment, Cd(ii) migrates along food chains and ultimately enters the human body, inducing severe health outcomes such as cancer, renal failure, and osteoporosis.^6^ Therefore, the treatment of Cd(ii)-containing AMD is necessary and urgent. Owing to its cost-effectiveness, operational simplicity, and high efficacy, adsorption stands out as a preferred method for the decontamination of heavy metals from wastewater.^7,8^ The selection of an appropriate adsorbent remains a decisive factor governing the overall treatment performance for acidic Cd(ii)-bearing wastewater.

For adsorption-based remediation, successful contaminant removal relies on the use of adsorbents exhibiting strong affinity toward target species and high uptake capacity.^9^ Various carbonaceous substrates, notably carbon nanotubes, graphene, and biochar, have shown great applicability as adsorbents for remediating heavy metal pollution, owing to their abundance of oxygen-containing functionalities, developed pore networks, and favorable physicochemical stability.^10^ In particular, biochar offers a distinct cost advantage, enabling greater potential for practical deployment in heavy-metal wastewater treatment.^4,7^ Nevertheless, pristine biochar frequently suffers from limited adsorption capacity and insufficient selectivity toward metal ions.^11^ Increasing the abundance of oxygen-containing functional groups has been proposed as an effective strategy to enhance metal binding on biochar surfaces. Wu *et al.*^12^ reported that HCl-washed biochar exhibited elevated contents of carboxyl and phenolic hydroxyl groups relative to pristine biochar. Acid oxidation has also been applied to sludge-derived biochar. Acid modification increased carboxyl and hydroxyl functionalities, whereas decreases in specific surface area, pore volume, and average pore size were observed after treatment.^13^ Oxidation using 15% H_2_O_2_ and mixed-acid activation with HNO_3_/H_2_SO_4_ similarly increased the abundance of carboxyl groups, and H_2_O_2_ oxidation was reported to be more effective in enriching surface carboxyl functionalities, accompanied by improved Cd(ii) adsorption.^14^ Beyond oxidation-based treatments, Fe-based modification has attracted extensive attention due to the specific affinity of iron oxides toward heavy metals, and Fe–O moieties can immobilize Cd(ii) effectively *via* inner-sphere complexation.^7,15^ Phosphorus modification not only enhances pore development and specific surface area through chemical etching, but also enables the formation of sparingly soluble phosphate precipitates with heavy metals, such as cadmium phosphate, representing a direct and highly stable immobilization pathway.^3^ Despite these advantages, studies addressing P/Fe co-modified sludge biochar for Cd(ii) removal from AMD remain limited, and the associated synergistic mechanisms are insufficiently resolved. While numerous studies have explored biochar modification for heavy metal removal, this work distinguishes itself by employing a synergistic P/Fe co-impregnation strategy using FeCl_3_ and KH_2_PO_4_. This approach aims to transcend the limitations of single-component modification by integrating the chemical etching and precipitation capacity of phosphate with the complexation activity of iron oxides, thereby creating a robust dual-mode immobilization network for Cd(ii).

In this work, a P/Fe co-impregnation modification strategy using FeCl_3_ and KH_2_PO_4_ was applied to sludge-derived biochar. The approach aims to integrate the pore-forming and precipitation capacity of P with the complexation activity of Fe, thereby constructing an engineered biochar (P–Fe@SBC) featuring high specific surface area and abundant reactive sites. The objectives of this study include: (1) systematic characterization of physicochemical property evolution before and after modification; (2) evaluation of the effect of solution pH and coexisting ions on Cd(ii) sorption performances; (3) elucidation of synergistic removal mechanisms through integrated kinetic, thermodynamic, and spectroscopic analyses. The findings are expected to provide both mechanistic understanding and technical support for efficient Cd(ii) remediation in AMD.

## 2 Material and method

### 2.1 Reagent and material

Cadmium nitrate tetrahydrate [Cd(NO_3_)_2_·4H_2_O], nitric acid (HNO_3_), sodium hydroxide (NaOH), ferric chloride hexahydrate (FeCl_3_·6H_2_O), and potassium dihydrogen phosphate (KH_2_PO_4_) were of analytical grade and purchased from Sinopharm Chemical Reagent Co., Ltd (Shanghai, China). Cd(ii) stock solutions at desired concentrations were prepared by dissolving predetermined masses of Cd(NO_3_)_2_·4H_2_O in deionized water.

Dewatered excess sludge was collected from a municipal wastewater treatment plant in Lanzhou, China. The sludges were air-dried under ventilated conditions for 7 days, followed by crushing into small granules. The granulated sludge was further dried at 60 °C to constant mass, then ground into powder and stored for subsequent use.

### 2.2 Preparation of biochar materials

FeCl_3_·6H_2_O (1.0 g) and KH_2_PO_4_ (1.0 g) were dissolved in 100 mL of deionized water under stirring until complete dissolution. Dried sludge powder (5.0 g) was added to the mixed solution. The suspension was stirred at 150 rpm for 24 h to ensure sufficient contact between modifiers and sludge particles. The mixture was dried overnight at 60 °C to remove water. The dried solid was pyrolyzed in a tubular furnace under N_2_ (flow rate: 10 mL min^−1^) at a heating rate of 20 °C min^−1^ to 800 °C, held for 2 h. After pyrolysis, the sample was naturally cooled to room temperature. The obtained biochar was repeatedly washed with deionized water to remove surface ash and unreacted ions until the filtrate pH approached neutrality, then dried at 60 °C. The product was denoted as P–Fe@SBC. Control materials: two control biochars were prepared to elucidate the roles of the modifiers. (1) Pristine sludge biochar (SBC): dried sludge powder without chemical impregnation was pyrolyzed under identical conditions and denoted as SBC.

(2) Fe-modified sludge biochar (Fe@SBC): prepared following the same procedure as P–Fe@SBC, except that only 1.0 g FeCl_3_·6H_2_O was added to the impregnation solution without KH_2_PO_4_. All subsequent steps remained unchanged, and the product was denoted as Fe@SBC.

### 2.3 Batch adsorption experiments

Cd(ii) solution (20 mL, 10 mg L^−1^) and adsorbent (0.2 g L^−1^) were added to 50 mL of polypropylene centrifuge tubes. The initial pH was adjusted using 0.1 M of HNO_3_/NaOH. The mixtures were shaken at 150 rpm and 25 °C for 300 min in a rotary shaker. The effects of initial pH (2.0–7.0), humic acid (0.5–10 mg L^−1^), and coexisting ions (0–0.1 mol L^−1^) on Cd(ii) removal were investigated. After reaction, suspensions were filtered through 0.45 µm nylon membrane filters. Residual Cd(ii) concentrations were determined by atomic absorption spectrophotometry (Shimadzu, Japan; AA-6300). All experiments were conducted in triplicate. Mean values and standard deviations were calculated.

Adsorption kinetics were investigated at predetermined time intervals (5–1200 min) using the same number of centrifuge tubes as sampling points. Each tube received 20 mL of Cd(ii) solution (10 mg L^−1^) and adsorbent at 0.2 g L^−1^. The solution pH was adjusted to 5.0 using 0.1 M HNO_3_/NaOH. The suspensions were shaken at 150 rpm and 25 °C for 1200 min. At designated times, the suspensions were filtered, and residual Cd(ii) concentrations in the filtrates were determined.

Adsorption isotherms were obtained by contacting 20 mL Cd(ii) solutions with initial concentrations of 10–200 mg L^−1^ with adsorbent at 0.2 g L^−1^ in 50 mL centrifuge tubes. The solution pH was adjusted to 5.0 using 0.1 M HNO_3_/NaOH. The mixtures were shaken at 150 rpm and 25 °C for 1200 min, then filtered. Residual Cd(ii) concentrations in the filtrates were measured.

### 2.4 Reusability experiments

A Cd(ii) solution (10 mg L^−1^) was mixed with adsorbent (0.2 g L^−1^), and the pH was adjusted to 5.0. The mixtures were shaken at 150 rpm and 25 °C for 1200 min, followed by filtration and determination of residual Cd(ii) concentration. The spent adsorbent was regenerated by shaking with 0.1 M NaOH at 25 °C for 24 h. The regenerated adsorbent was collected by filtration, rinsed three times with deionized water, dried at 80 °C for 24 h, then reused in the subsequent adsorption cycle under identical conditions.

## 3 Result and discussion

### 3.1 Physicochemical properties

**Fig. 1:**
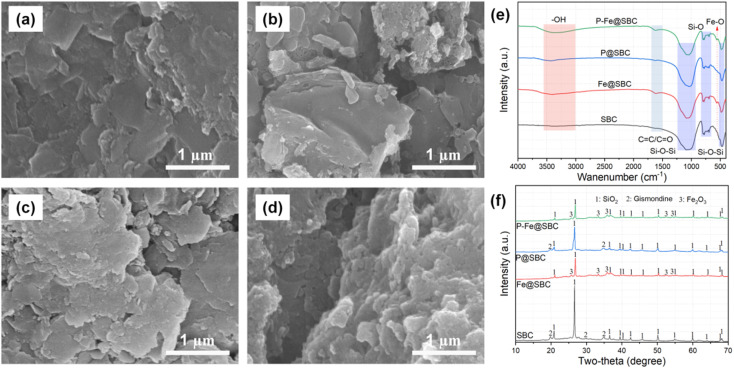
Morphology ((a): SBC, (b): Fe@SBC, (c): P@SBC and (d): P–Fe@SBC), FTIR (e), and XRD (f) analysis of different adsorbents.

As shown in Fig. 1a, pristine SBC exhibited a compact blocky/lamellar morphology with a relatively smooth surface, limited visible pores, no obvious deposits, indicating a dense carbon-mineral matrix. After Fe modification (Fig. 1b), Fe@SBC displayed a markedly roughened surface with irregular etching traces and fine granular protrusions on the carbon framework, implying successful loading of Fe-containing phases and concurrent surface activation. P–Fe@SBC (Fig. 1c) was densely covered by aggregated particulates and amorphous precipitates, generating a highly rough, loosely packed porous texture. Such a hierarchically roughened surface increases interfacial contact area and enriches accessible reactive sites, favoring Cd(ii) uptake. The surface elemental compositions of the various biochar samples were quantitatively characterized *via* EDS spectra (Fig. S1a–d). The results indicate that the pristine biochar (SBC) is predominantly composed of C, O, Si, and Al, with negligible amounts of Fe (1.47 wt%) and P (0.15 wt%). Following iron modification, the Fe content in Fe@SBC increased to 16.38 wt%, clearly confirming the successful loading of iron oxides or iron-containing species onto the biochar matrix. Similarly, the P content in P@SBC rose from 0.15 wt% to 4.24 wt%, demonstrating the effective incorporation of phosphate functional groups. For the co-modified biochar (P–Fe@SBC), both Fe (7.14 wt%) and P (3.61 wt%) remained at relatively high levels, suggesting a synergistic loading of iron and phosphorus on the biochar surface. Notably, a distinct increasing trend in O content was observed as the modification progressed, rising from 31.69 wt% in SBC to a maximum of 43.46 wt% in P–Fe@SBC. This substantial enrichment of oxygen is primarily attributed to the joint contribution of oxygen-containing iron species and phosphate moieties, which not only alters the surface chemistry of the material but also provides abundant oxygen-containing active sites for the subsequent high-efficiency removal of Cd(ii).

The specific surface area and pore characteristics of SBC, Fe@SBC, P@SBC, and P–Fe@SBC were investigated *via* N_2_ adsorption–desorption isotherms and pore size distribution curves. As shown in Fig. S2a, all samples exhibited typical Type IV isotherms with H3 hysteresis loops, indicating the presence of abundant mesoporous structures.^11,16^ As displayed in Fig. S2b, the pore size distribution curves further confirmed the mesoporous nature, with pore widths mainly distributed between 2 and 50 nm.^12,17^ Notably, the N_2_ adsorption capacity increased significantly after modification (especially for P–Fe@SBC), which was attributed to the optimized pore structure and increased accessibility of active sites provided by the introduction of Fe and P species. Textural parameters (Table 1) further supported the above observations. Pristine SBC presented a low specific surface area (SSA, 18.632 m^2^ g^−1^) and a small total pore volume (0.183 × 10^−2^ cm^3^ g^−1^), plausibly associated with pore blockage by abundant inorganic ash inherent to sludge feedstocks. Upon Fe incorporation, SSA and pore volume of Fe-SBC increased to 86.597 m^2^ g^−1^ and 0.072 cm^3^ g^−1^, respectively. The improvement can be attributed to *in situ* formation of iron oxide particles within the carbonaceous matrix, providing a rigid scaffold that mitigates pore collapse, catalytic cracking that suppresses tar deposition inside pores. Notably, the phosphate-only modification (P@SBC) also significantly enhanced the textural properties, with the SSA and pore volume rising to 106.147 m^2^ g^−1^ and 0.116 cm^3^ g^−1^, respectively. This improvement suggested that the introduction of phosphate species effectively initiated chemical etching and promoted the development of a more open carbon framework.^6^ P/Fe co-modification yielded the most developed porosity. SSA reached 137.915 m^2^ g^−1^. Total pore volume increased to 0.218 cm^3^ g^−1^. Phosphate introduction provided strong chemical activation and etching during carbonization, promoted volatile release, etched carbon frameworks, induced a more abundant multi-level pore network. The average pore diameter expanded from 9.68 nm to 15.98 nm, indicating a typical mesoporous architecture.^11,18^ Enlarged mesoporous channels facilitate rapid diffusion of Cd(ii) toward internal active sites, improving adsorption kinetics.

**Table 1:** Pore structure parameters of SBC, Fe@SBC, P@SBC and P–Fe@SBC

	SBC	Fe@SBC	P@SBC	P–Fe@SBC
Specific surface area (m^2^ g^−1^)	18.632	86.597	106.147	137.915
Pore volume (cm^3^ g^−1^)	0.183 × 10^−2^	0.072	0.116	0.218
Average pore diameter (nm)	9.681	12.934	13.742	15.981

FTIR spectra (Fig. 1d) revealed pronounced evolution of surface functional groups after modification. The broad band at 3410 cm^−1^ was assigned to –OH stretching vibration, with an intensity sequence of P–Fe@SBC > Fe@SBC > P@SBC > SBC.^9,19^ The enhancement was associated with Fe loading that introduces Fe–OH moieties, phosphate co-modification that provides additional P–OH groups.^20^ Enriched hydroxyl groups favor Cd(ii) capture *via* ion exchange and surface complexation. The band near 1620 cm^−1^ corresponded to aromatic C=C skeletal vibration or C=O stretching from oxygen-containing groups.^10^ SBC showed a weak response at this region, suggesting limited surface functionalities or a low aromatization degree. A distinct peak at approximately 1620 cm^−1^ emerged after modification, with higher intensity for P–Fe@SBC than Fe@SBC and P@SBC, indicating promoted aromatization or increased abundance of oxygenated groups (*e.g.*, carboxyl/carbonyl) induced by Fe/P-assisted catalytic effects during pyrolysis. A characteristic Fe–O vibration band appeared at 560 cm^−1^ in Fe@SBC and P–Fe@SBC, confirming iron-oxide-related structures.^10,20^ Strong silicate bands were observed in all samples, consistent with the high mineral content of sludge. The prominent absorption peaks observed at 1030–1080 cm^−1^ and ∼470 cm^−1^ were ascribed to the antisymmetric stretching vibrations of Si–O–Si, while the band around 780 cm^−1^ was identified as symmetric Si–O stretching.^15^

XRD patterns (Fig. 1e) indicated the presence of a stable silicate-rich mineral framework. Quartz (SiO_2_, JCPDS No. 46-1045) reflections were detected for all samples, consistent with an intact mineral skeleton after impregnation and pyrolysis.^15,16^ In SBC and P@SBC, peaks attributable to gismondine (JCPDS No. 20-0452) were also observed.^16^ These reflections disappeared in Fe@SBC and P–Fe@SBC, suggesting dissolution or structural disruption of Ca–Al–silicate phases under the acidic FeCl_3_ impregnation environment, or Fe-driven mineral transformation. New diffraction peaks assigned to hematite (α-Fe_2_O_3_, JCPDS No. 33-0664) appeared after modification, providing evidence for the precipitation of crystalline iron oxides on the biochar surface.^10,20^ Notably, no distinct crystalline phosphate phases were identified for P@SBC and P–Fe@SBC. Phosphorus may exist in an amorphous form with high dispersion, or as low-crystallinity Fe/P surface complexes associated with iron oxides. The Raman spectra (Fig. S2c) were employed to investigate the structural evolution and defect density of the biochar carbon skeleton. All samples exhibited two characteristic bands at approximately 1350 cm^−1^ (D-band) and 1590 cm^−1^ (G-band), representing the disordered/amorphous carbon structures and the in-plane vibrations of sp^2^-hybridized carbon atoms, respectively.^17^ The *I*_D_/*I*_G_ ratio, a key indicator of structural defects, showed a continuous decline from 2.18 (SBC) to 2.03 (Fe@SBC), 1.76 (P@SBC), and finally to 1.29 (P–Fe@SBC). This reduction in the *I*_D_/*I*_G_ ratio suggested that the co-modification with iron and phosphate promoted the rearrangement of the carbon framework, leading to an increased degree of graphitization and structural ordering.^11^ The enhanced structural integrity of P–Fe@SBC was conducive to maintaining chemical stability during the adsorption process, further supporting its superior performance in environmental remediation applications.^12^

### 3.2 Batch adsorption experiments

#### 3.2.1 Initial solution pH

**Fig. 2:**
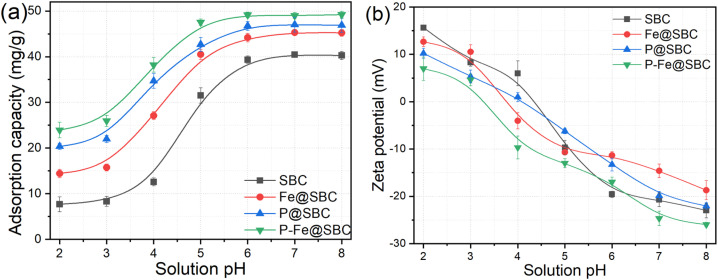
Removal performance (a) and zeta potential (b) of adsorbent for Cd(ii) at different initial pH.

Fig. 2a illustrated the Cd(ii) removal efficiencies of biochar across varying initial pH. Increasing pH from 2.0 to 5.0 led to a rapid increase in adsorption capacity for all adsorbents. At pH > 5.0, the increase became marginal; adsorption capacities approached 40 mg g^−1^ (SBC), 45 mg g^−1^ (Fe@SBC), 47 mg g^−1^ (P@SBC), 49 mg g^−1^ (P–Fe@SBC). Cd(ii) speciation within the investigated pH range remained dominated by cationic Cd^2+^, indicating a key role of surface charge in governing removal.^11^ The zeta potential profiles (Fig. 2b) exhibited a charge reversal from positive to negative across the pH range of 2–8, indicating a switch from positively charged to negatively charged surfaces. The points of zero charge (pH_PZC_) were 4.38 (SBC), 3.72 (Fe@SBC), 4.14 (P@SBC), 3.32 (P–Fe@SBC). At pH < pH_PZC_, positively charged surfaces imposed electrostatic repulsion toward Cd^2+^; abundant H^+^ competitively occupied reactive sites.^2,8^ At pH > pH_PZC_, increasing surface electronegativity enhanced electrostatic attraction toward Cd^2+^, improving uptake.^9,21^ These results supported electrostatic interaction as a contributing pathway for Cd(ii) removal.

#### 3.2.2 Coexisting ions

**Fig. 3:**
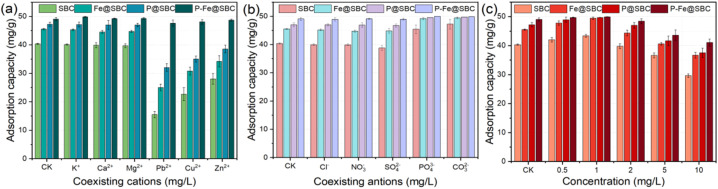
Effects of co-existing cation (a), anion (b), and humic acid (c) on Cd(ii) removal.

The effects of coexisting ions on Cd(ii) removal are presented in Fig. 3a. Alkali/alkaline-earth cations (K^+^, Ca^2+^, Mg^2+^) caused negligible changes in Cd(ii) adsorption by SBC, Fe@SBC, P@SBC, and P–Fe@SBC, indicating limited competition under these conditions. In contrast, Pb^2+^, Cu^2+^, and Zn^2+^ reduced Cd(ii) adsorption, demonstrating competitive uptake. Inhibition followed the order Pb^2+^ > Cu^2+^ > Zn^2+^, consistent with differences in electronegativity [Pb (2.33) > Cu (1.90) > Cd (1.69) > Zn (1.65)].^11,14^ More electronegative metal ions, especially Pb^2+^, preferentially formed stronger inner-sphere surface complexes, occupying oxygen-containing functional groups and modification-derived sites (Fe–O, P–O), generating pronounced site-masking effects toward Cd(ii).^15,22^ Although the presence of coexisting heavy metals like Pb^2+^ inhibited Cd(ii) uptake due to site-masking effects, this challenge was addressed in practical remediation through several strategies. First, the ultra-high maximum capacity of P–Fe@SBC (328.95 mg g^−1^) provided a substantial ‘site buffer,’ which allowed for efficient Cd(ii) removal even when some sites were occupied by Pb^2+^. Second, the synergistic combination of phosphate-driven precipitation and iron-oxide complexation offered a diverse array of reactive domains, some of which exhibited varying affinities for different metal ions. In engineering practice, increasing the adsorbent dosage or employing a multi-stage adsorption process effectively mitigated the competitive inhibition by Pb^2+^, ensuring that Cd(ii) concentrations in complex acid mine drainage (AMD) met discharge standards.

As shown in Fig. 3b, coexisting anions (Cl^−^, NO_3_^−^, SO_4_^2−^) exerted only minor influence, implying that Cd(ii) binding was dominated by specific inner-sphere complexation rather than ionic-strength-sensitive outer-sphere adsorption. In contrast, PO_4_^3−^ and CO_3_^2−^ enhanced adsorption capacity, indicating a coupled removal pathway. These anions reacted with Cd(ii), inducing formation of sparingly soluble precipitates on the adsorbent surface.^11^ The surface served as nucleation and growth sites, transforming the process into an “adsorption-precipitation” dual-mode removal.

#### 3.2.3 Humic acid

Humic acid (HA), a ubiquitous dissolved organic matter component, significantly regulates heavy-metal behavior. The influence of HA concentration on Cd(ii) removal is shown in Fig. 3c. At low HA levels (0–1 mg L^−1^), adsorption capacity increased. HA adsorbed on biochar acted as a ligand bridge, promoting formation of stable ternary surface complexes [biochar–HA–Cd(ii)], providing additional binding domains. At higher HA levels (up to 10 mg L^−1^), adsorption was strongly inhibited. Excess dissolved HA formed highly stable, soluble Cd–HA complexes in the aqueous phase, limiting Cd(ii) transport toward the sorbent surface.^17,23^ Macromolecular HA aggregation on biochar surfaces likely blocked pores, introducing steric hindrance and deactivating internal sites.^23^

### 3.3 Adsorption behaviors

#### 3.3.1 Kinetics

**Fig. 4:**
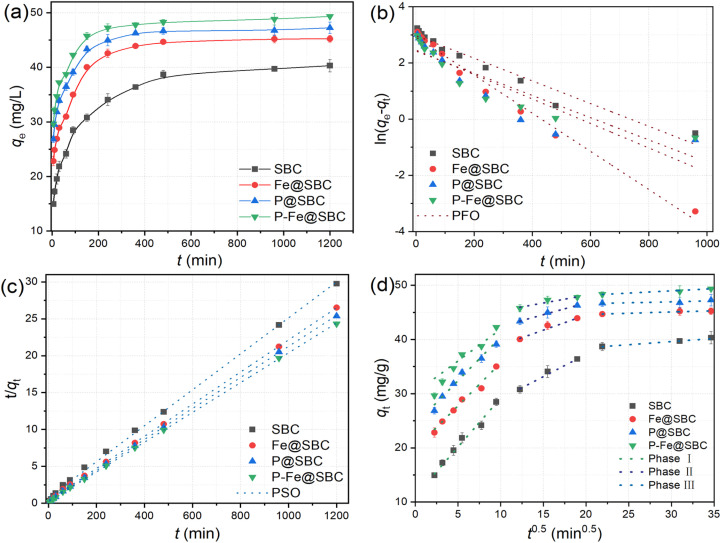
Effects of contact time on removing Cd(ii) (a) and the fitted pseudo-first-order (b), pseudo-second-order (c), and intra-particle diffusion (d) model.

As shown in Fig. 4a, the three adsorbents exhibited comparable kinetic profiles for Cd(ii) uptake over 0–1200 min. A rapid increase occurred at the initial stage, followed by a gradual rise toward equilibrium. The initial fast uptake was attributed to the abundance of available surface sites and a steep concentration gradient, enabling prompt Cd(ii) binding.^5,24^ With prolonged contact time, progressive occupation of reactive sites reduced the number of accessible sites, resulting in a slower increase in adsorption capacity. Equilibrium uptake stabilized at approximately 40, 45, 47, and 49 mg g^−1^ for SBC, Fe@SBC, P@SBC, and P–Fe@SBC, respectively.

To interpret the adsorption process, three kinetic models were applied to analyze the experimental data: the PFO model (Fig. 4b), the PSO model (Fig. 4c), and the IPD model (Fig. 4d). In Table 2, the PSO model provided the best fit, with an *R*^2^ value of 0.999, exceeding those obtained from the PFO model (*R*^2^ = 0.78–0.94). The equilibrium adsorption capacities calculated from the PSO model (*q*_e,cal_) showed closer agreement with the experimental values (*q*_e,exp_). The results indicated that Cd(ii) removal involved combined physical and chemical contributions, with chemisorption playing a dominant role.^25,26^ In this context, Cd(ii) immobilization was primarily associated with electron sharing or exchange between Cd(ii) and reactive surface moieties, forming relatively stable chemical bonds.^22,27^

**Table 2:** Fitted parameter for sorption kinetics of Cd(ii) removal

	Parameter	SBC	Fe@SBC	P@SBC	P–Fe@SBC
PFO	*q*_e,exp_ (mg g^−1^)	40	45	47	49
	*q*_e,cal_ (mg g^−1^)	19.571	18.579	11.700	11.263
	*k*_1_ (1/min)	0.403 × 10^−2^	0.678 × 10^−2^	−0.435 × 10^−2^	0.395 × 10^−2^
	*R* ^2^	0.951	0.988	0.782	0.832
PSO	*q*_e,cal_ (mg g^−1^)	41.051	45.935	47.596	49.579
	*k*_2_ (g mg^−1^ min^−1^)	0.077 × 10^−2^	0.126 × 10^−2^	0.177 × 10^−2^	0.187 × 10^−2^
	*R* ^2^	0.999	0.999	0.999	0.999
IPD	*K*_d1_ (mg g^−1^ min^−1/2^)	1.806	0.821	0.607	0.115
	*C*_1_	11.138	20.838	24.369	36.164
	*R*_1_^2^	0.991	0.996	0.978	0.987
	*K*_d2_ (mg g^−1^ min^−1/2^)	1.580	0.555	0.435	0.047
	*C*_2_	19.866	33.398	38.046	43.666
	*R*_2_^2^	0.994	0.988	0.998	0.975
	*K*_d3_ (mg g^−1^ min^−1/2^)	1.171	0.286	0.035	0.008
	*C*_3_	30.182	42.445	45.897	46.502
	*R*_3_^2^	0.937	0.938	0.958	0.990

Mass-transfer analysis based on the IPD model suggested a three-stage uptake behavior for SBC, Fe@SBC, P@SBC, and P–Fe@SBC: a rapid sorption stages (0–90 min), a slower sorption stages (150–360 min), and an equilibrium stages (480–1200 min). During the initial stage, a significant concentration gradient between the bulk phase and the adsorbent surface acted as a driving force, accelerating the transport of Cd(ii) through the boundary layer to the external surface, consistent with film diffusion.^11^ In Stage II, depletion of surface sites promoted the diffusion of surface-associated Cd(ii) into internal pores, corresponding to intraparticle diffusion and subsequent binding to internal sites.^19,24^ Stage III represented an approach to dynamic equilibrium. According to Table 2, the IPD model yielded *R*^2^ values above 0.90 for all materials. The intercept terms (*C*_1_, *C*_2_, and *C*_3_) were non-zero, suggesting that intraparticle diffusion was not the only rate-controlling step and that multiple transport resistances contributed.^11,28^ The boundary-layer diffusion constant (*K*_d1_) in Stage I was substantially higher than those in Stages II and III, supporting film diffusion as the primary rate-controlling step for removing Cd(ii) under the tested conditions.^26,28^

#### 3.3.2 Adsorption isotherms

**Fig. 5:**
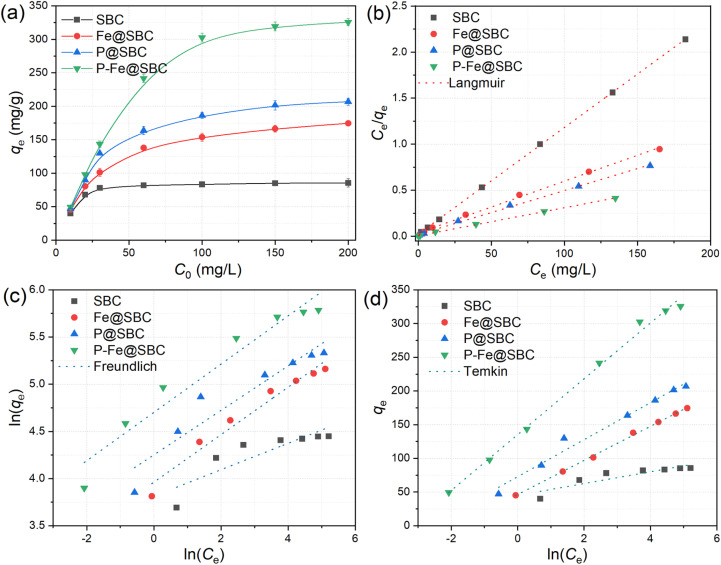
Effects of Cd(ii) concentrations on removing performance (a) and the fitted Langmuir (b), Freundlich (c), and Temkin (d) isotherm model.

In Fig. 5a, increasing the Cd(ii) concentrations from 10 to 200 mg L^−1^ produced a rapid rise in adsorption capacity at low concentrations, followed by a plateau at higher concentrations. The maximum uptake capacities reached 86 mg g^−1^ for SBC, 175 mg g^−1^ for Fe@SBC, 207 mg g^−1^ for P@SBC, 326 mg g^−1^ for P–Fe@SBC. At low Cd(ii) levels, abundant accessible binding sites and surface functional groups support efficient Cd(ii) capture. Progressive site occupation occurs with increasing Cd(ii) concentration, leading to saturation of available sites and an approach to the maximum capacity.^16,27^

Isotherm models are commonly used to interpret equilibrium mechanisms, estimate adsorption capacity, and describe intrinsic adsorption features, providing a quantitative basis for evaluating adsorbent performance. The fitting results of the Langmuir (Fig. 5b), Freundlich (Fig. 5c) and Temkin (Fig. 5d) models are presented in Table 3. The Langmuir model provided excellent fits for Cd(ii) adsorption on SBC, Fe@SBC, P@SBC, and P–Fe@SBC, with correlation coefficients exceeding 0.99, indicating a predominance of monolayer adsorption.^11^ Langmuir-derived theoretical maximum capacities were 86 mg g^−1^ (SBC), 178 mg g^−1^ (Fe@SBC), 210 mg g^−1^ (P@SBC), 329 mg g^−1^ (P–Fe@SBC). The Langmuir separation factor (RL, Fig. S1) remained within 0–1, indicating favorable adsorption over the investigated concentration range. Lower RL values for P–Fe@SBC relative to SBC, P@SBC, and Fe@SBC indicated stronger affinity and higher feasibility for Cd(ii) removal in aqueous solution.^11^

**Table 3:** Isotherm fitting parameters for Cd(ii) removal

		SBC	Fe@SBC	P@SBC	P–Fe@SBC
Langmuir	*q*_max_ (mg g^−1^)	86.356	177.809	209.644	328.947
	*K*_L_ (L mg^−1^)	0.078	0.151	0.242	0.469
	*R* ^2^	0.999	0.998	0.998	0.999
Freundlich	*K*_f_ (mg^1−*n*^ L^*n*^ g^−1^)	45.334	52.563	70.238	110.167
	*n*	7.137	3.972	4.244	3.910
	*R* ^2^	0.748	0.966	0.879	0.941
Temkin	*A*_T_ (1/g)	175.874	6.252	14.193	26.242
	*B*_T_ (kJ mol^−1^)	8.747	25.305	27.470	41.424
	*R* ^2^	0.774	0.908	0.974	0.923

To further evaluate the adsorption performance of P–Fe@SBC, its maximum Cd(ii) adsorption capacity (*q*_max_) was compared with various previously reported biochar-based adsorbents (Table S1). The *q*_max_ of P–Fe@SBC (326 mg g^−1^) is significantly higher than that of many reported materials, such as iron and silicon modified biochar (31.66 mg g^−1^),^20^ Chitosan@coconut shell-derived biochar (63.88 mg g^−1^),^11^ HCl-modified biochar (68.22 mg g^−1^),^12^ EDTA functionalized Mg/Al hydroxides modified biochar (204.53 mg g^−1^),^9^ Cysteine-grafted magnesium-modified biochar (223.7 mg g^−1^),^26^ hydroxyl-functionalized Fe/Ni-biochar (229.52 mg g^−1^)^5^ and multifunctional magnetic biochar (292 mg g^−1^).^17^ This superior performance was primarily attributed to the synergistic effect of P and Fe co-modification, which not only provided high specific surface area but also enriched the surface with multiple active binding sites, including iron-oxide groups and phosphate moieties. The maximum Cd(ii) adsorption capacity achieved by P–Fe@SBC (328.95 mg g^−1^) significantly outperformed most recently reported sludge-derived adsorbents (Table S1). This exceptional performance validated that the co-modification did not merely aggregate individual benefits but induced a synergistic enhancement of the interfacial chemistry.

### 3.4 Potential Cd(ii) removal mechanisms

**Fig. 6:**
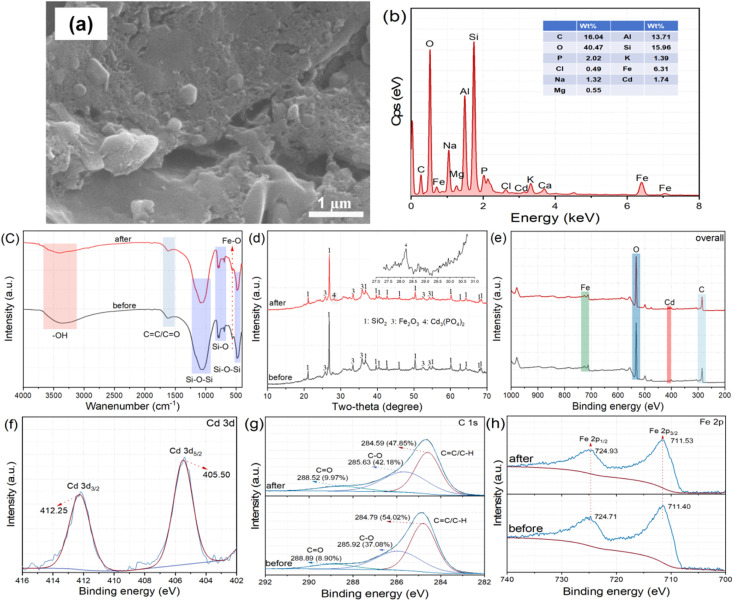
Morphology (a), surface elemental distribution (b), FTIR spectra (c), XRD patterns (d), XPS survey scan (e), and high-resolution XPS spectra of Cd 3d (f), C 1s (g), and Fe 2p (h) of P–Fe@SBC after Cd(ii) adsorption.

SEM images (Fig. 6a) show a heterogeneous and rough surface after adsorption, accompanied by abundant irregular granular aggregates. Dispersed fine particles are visible, attributable to Cd-bearing precipitates or surface complexes. The Cd signal is clearly detected in the EDS spectrum (Fig. 6b). Quantitative analysis gives a Cd content of 1.74 wt%. Fe (6.31 wt%) and P (2.02 wt%) remain at high levels after adsorption, indicating good chemical stability of the functional components. High contents of Si (15.96 wt%) and Al (13.71 wt%) further confirm the mineral/ash-rich nature of sludge-derived biochar. To further substantiate the multi-pathway mechanism, we have supplemented the analysis of elemental evolution after adsorption. The EDS results of P–Fe@SBC following the reaction showed a distinct decline in the weight percentages of K, Na, Ca, and Mg. This provided direct evidence for the involvement of ion exchange between these naturally occurring mineral cations and aqueous Cd^2+ 25^.

FTIR spectra (Fig. 6c) reveal pronounced band shifts after Cd(ii) uptake. The –OH band shifts from 3410 to 3420 cm^−1^ with decreased intensity, supporting deprotonation of surface hydroxyls and subsequent coordination with Cd(ii), accompanied by a change in force constants.^24,26^ The band assigned to aromatic C=C vibration or C=O stretching shifts from 1620 to 1630 cm^−1^, implying electron donation from the oxygen-containing group to the empty orbitals of Cd(ii) or π electrons of aromatic structures.^4,26,28^ The asymmetric Si–O–Si stretching vibration bands, which typically appeared with high intensity in the range of 1030–1080 cm^−1^ due to significant changes in the dipole moment, remained nearly unchanged after Cd(ii) uptake.^23^ A slight shift of the Si–O symmetric stretching band from 780 to 785 cm^−1^ reflected subtle local environmental perturbations near active sites.^15^ The Fe–O band shifts markedly from 560 to 570 cm^−1^, providing direct evidence for involvement of iron-oxide sites and formation of inner-sphere Fe–O–Cd complexes.^10,20^

XRD patterns (Fig. 6d) before and after adsorption show stable diffraction peak positions and intensities for SiO_2_ and α-Fe_2_O_3_, indicating that the silicate skeleton and the loaded iron-oxide phase remain structurally intact, without notable dissolution or phase transformation.^10,23^ To further clarify the precipitation products, an enlarged view of the characteristic Cd_3_(PO_4_)_2_ (JCPDS No. 14-0131) reflections was provided in the inset of Fig. 6d. New reflections assigned to Cd_3_(PO_4_)_2_ appear after adsorption, confirming chemical precipitation as a key immobilization pathway. Phosphate species introduced by modification (PO_4_^3−^/HPO_4_^2−^) act as precipitation agents, reacting with captured Cd^2+^ to induce *in situ* formation of stable cadmium phosphate on the biochar surface, enabling persistent fixation.^12,23^

XPS survey spectra (Fig. 6e) show dominant signals at 285 eV (C 1s), 532 eV (O 1s), 711 eV (Fe 2p) for both samples. After adsorption, new Cd 3d signals appear at 405 eV and 412 eV, confirming Cd enrichment on the P–Fe@SBC surface. High-resolution Cd 3d spectra (Fig. 6f) display two distinct peak at 405.50 eV and 412.25 eV, assigned to Cd 3d_5/2_ and Cd 3d_3/2_, respectively. C 1s spectra (Fig. 6g) are deconvoluted into three components: graphitic/aliphatic carbon (C=C/C–H), singly bonded O carbon (C–O), doubly bonded O carbon (C=O).^2,8^ After adsorption, binding energies decrease from 284.79, 285.92, 288.89 eV to 284.59, 285.63, 288.52 eV, consistent with coordination of oxygen atoms to Cd(ii) and redistribution of electron density, yielding C–O–Cd or C=O–Cd surface complexes.^12,23^ The relative fraction of C–O increases from 37.08% to 42.18% and C=O increases from 8.90% to 9.97%, supporting oxygenated groups as major reactive sites *via* surface complexation.^10,18^ The shift and attenuation of the C=C component further suggest participation of cation–π interactions.^12^ Fe 2p high-resolution spectra (Fig. 6h) provide additional evidence for Fe-site involvement. Before adsorption, Fe 2p_3/2_ and Fe 2p_1/2_ at 711.40 eV and 724.71 eV match Fe(iii) oxides, consistent with α-Fe_2_O_3_ on the biochar surface. After adsorption, the peaks shift to 711.53 eV and 724.93 eV, indicating decreased electron density around Fe, attributable to Fe–O groups coordinating with Cd(ii) and forming Fe–O–Cd linkages.^10,18^ Iron oxides function as active binding centers rather than inert loadings.

Overall, Cd(ii) removal by P–Fe@SBC involves a coupled physicochemical process. Electrostatic attraction accelerates interfacial enrichment, driven by negatively charged sites created by deprotonated oxygen-containing groups (R–O^−^, –COO^−^) and phosphate species. Phosphate components induce *in situ* crystallization of sparingly soluble Cd_3_(PO_4_)_2_. Oxygenated functionalities (C–O, C=O) and iron-oxide sites (Fe–O) act as electron-donating ligands, generating stable inner-sphere complexes (*e.g.*, Fe–O–Cd). The aromatic carbon framework contributes *via* cation–π interaction. Mineral-associated exchangeable cations contribute *via* ion exchange. These pathways collectively account for the superior Cd(ii) immobilization performance.

### 3.5 Reusability

Across five consecutive cycles (Fig. S4a), Cd(ii) removal by SBC, Fe@SBC, and P–Fe@SBC exhibited a gradual decline. After the fifth cycle, adsorption capacities were 18.95 mg g^−1^ (SBC), 107.67 mg g^−1^ (Fe@SBC), 286.97 mg g^−1^ (P–Fe@SBC), corresponding to decreases of 77.87%, 38.37%, 11.75%, respectively. The decline can be attributed to progressive loss or passivation of reactive sites after repeated adsorption–desorption. P–Fe@SBC showed only ∼10% reduction after five cycles, indicating strong structural stability and practical reusability. To evaluate the stability of P–Fe@SBC during the reuse cycles, the leaching ratios of Fe and P were monitored. As shown in Fig. S4b, the leaching percentages of Fe and P after the first cycle were 4.52% and 2.12%, respectively. With the increase of cycles, these percentages exhibited a continuous declining trend, reaching 0.97% for Fe and 0.52% for P by the fifth cycle. Such trace-level leaching percentages indicated a strong binding affinity between the modified components and the biochar carbon-mineral matrix. Despite this minor loss of active sites, P–Fe@SBC maintained a high adsorption capacity, losing only approximately 11.75% of its initial performance after five cycles.

## 4 Conclusion

P/Fe co-modified sludge biochar (P–Fe@SBC) was synthesized *via* FeCl_3_–KH_2_PO_4_ co-impregnation followed by pyrolysis. Iron oxide (α-Fe_2_O_3_) was successfully anchored on the biochar surface. Phosphate-driven chemical activation and etching promoted formation of a hierarchical pore network, yielding a high specific surface area (137.915 m^2^ g^−1^) and total pore volume (0.218 cm^3^ g^−1^), providing abundant transport channels and accessible sites for Cd(ii) capture. Cd(ii) adsorption followed the Langmuir isotherm model and the pseudo-second-order kinetic model. The maximum removal amount reached 328.95 mg g^−1^, far exceeding that of pristine biochar. Common alkali/alkaline-earth ions showed minor interference, indicating strong anti-interference capability. Reusability tests demonstrated high stability; adsorption capacity decreased by only ∼10% after five cycles. Mechanistic analyses indicate a synergistic removal network involving in electrostatic attraction, chemical precipitation, inner-sphere surface complexation, cation–π interaction, and ion exchange. This work provided a ‘waste-to-treat-waste’ strategy that transformed low-value sewage sludge into a high-performance adsorbent with superior capacity and stability, offering a novel perspective for the large-scale remediation of cadmium-contaminated acid mine drainage. Future studies will focus on optimizing the mass ratios of FeCl_3_ and KH_2_PO_4_ to further elucidate the quantitative relationship between precursor loading and the synergistic removal of heavy metals.

## Author contributions

Yunping Ji: conceptualization, methodology, investigation, software, funding acquisition, supervision, writing – original draft, writing – review & editing. Yarong Zhao: data curation, writing – original draft. Qingfeng Lv: writing – review & editing. Fei Gao: formal analysis, visualization. All authors have read and agreed to the published version of the manuscript.

## Conflicts of interest

The authors declare that they have no known competing financial interests or personal relationships that could have appeared to influence the work reported in this paper.

## Supplementary Material

RA-016-D5RA09939K-s001

## Data Availability

Data will be made available on request. Supplementary information (SI): supporting technical data and detailed methodology, including analytical methods: calculation formulas for adsorption kinetics (pseudo-first-order, pseudo-second-order, and intra-particle diffusion models) and adsorption isotherms (Langmuir, Freundlich, and Temkin models); characterization techniques: detailed parameters for SEM-EDS, FTIR, XRD, XPS, Raman spectroscopy, and N_2_ adsorption–desorption analysis; experimental results: figures and tables covering EDS spectra, pore size distributions, separation factors (*R*_L_), and the reusability/leaching stability of the adsorbents over multiple cycles; performance comparison: a comparative table (Table S1) summarizing the maximum adsorption capacity of P-Fe@SBC against other reported biochar-based adsorbents. See DOI: https://doi.org/10.1039/d5ra09939k.
