# Supplementary material for: Synergistic remediation of aqueous Cd(ii) by sewage sludge biochar via P/Fe co-impregnation
Source: RSC Adv. 2026 Feb 23;16(12):10679–88. doi: 10.1039/d5ra09939k (PMC12927708; doi:10.1039/d5ra09939k)
Supplement: RA-016-D5RA09939K-s001 [file RA-016-D5RA09939K-s001.pdf]

Supplementary Information (SI) for RSC Advances.
This journal is © The Royal Society of Chemistry 2026

Synergistic remediation of aqueous Cd(II) by sewage sludge biochar via P/Fe co-impregnation

Yunping Ji [a,b,], Yarong Zhao [c], Qingfeng lv [a], Fei Gao [d]*

[a] *School of Civil Engineering and Mechanics, Lanzhou University, Lanzhou 730000, China*

[b] *China Railway First Survey and Design Institute Group Co., Ltd., Lanzhou 730000, China*

[c] *Zhejiang Huancheng Environmental Protection Technology Co., Ltd., Hangzhou 310012, China*

[d] *China Railway 21st Bureau Group Co., Ltd., Lanzhou 730070, China*

**Corresponding author: Yunping Ji (shaopei13698@163.com)*

Text S1 Calculation Formulas and Adsorption Models

The sorption amount (q_e, mg/g) were computed according to Eq. (S1).

$$q_{e} = \frac{(C_0 - C_e) \times V}{m} \qquad \text{(S1)}$$

Where C_0 and C_e are the initial and final concentration of Cd(II) (mg/L), respectively; q_e is sorption amount (mg/g); V is the solution volume (mL); and m is the mass of adsorbent (mg).

Adsorption kinetics model: the pseudo-first-order model (Eq. S2), the pseudo-second-order model (Eq. S3), and intra-particle diffusion model (Eq. S4) were used for fitting analysis.

$$\ln(q_e - q_t) = \ln q_e - k_1 t \qquad \text{(S2)}$$

$$\frac{t}{q_t} = \frac{1}{q_e^2 k_2} + \frac{t}{q_e} \qquad \text{(S3)}$$

$$q_t = K_{id} t^{1/2} + C_i \qquad \text{(S4)}$$

Where q_e and q_t are the sorption capacity at the equilibrium time and time "t" time (mg/g), respectively; k_1 and k_2 represent sorption rate constant of the pseudo-first-order (1/min) and the pseudo-second-order (g/mg·min), respectively; K_{id} (mg/g·min$^{1/2}$) is rate constants of intra-particle diffusion, respectively; C_i is constant of the intra-particle diffusion model.

Adsorption isotherm model: the Langmuir model (Eq. S5), Freundlich model (Eq. S6), and Temkin model (Eq. S7) were used to fit these data.

$$\frac{C_e}{q_e} = \frac{1}{q_{max} K_L} + \frac{C_e}{q_{max}} \qquad RL = \frac{1}{1 + C_0 K_L} \qquad \text{(S5)}$$

$$\ln q_e = \ln K_F + \frac{1}{n} \ln C_e \qquad \text{(S6)}$$

$$q_e = B_T \ln A_T + B_T \ln C_e \qquad \text{(S7)}$$

Where q_e is the sorption capacity at equilibrium (mg/g); C_e is the concentration of Cd(II) at sorption equilibrium (mg/L); q_{max}, and K_L are the maximum sorption capacity of Cd(II) (mg/g), and Langmuir equilibrium constant (L/mg), respectively; K_f and n represent the Freundlich affinity coefficient (mg^{1-n}·L^n/g) and Freundlich constant related to the surface site heterogeneity, respectively; A_T (1/g) and B_T (kJ/mol) are Temkin constants.

Text S2 Characterization analysis

The N_2 adsorption-desorption isotherms were measured at 77 K using an automatic surface area and porosity analyzer (ASAP 2020 Plus HD88, Micromeritics, USA). Prior to analysis, all samples were degassed under vacuum at 150 °C for 6 h to remove moisture and impurities. The specific surface area (SSA) was calculated using the Brunauer-Emmett-Teller (BET) equation, while the pore size distribution and total pore volume were determined from the desorption branch of the isotherms using the Barrett-Joyner-Halenda (BJH) model. The microstructure of the biochars was tested by scanning electron microscopy (SEM-EDS, JSM-7500F, JEOL, Japan). The surface functional groups were analysed by Fourier transform infrared spectroscopy (FTIR, Nicolet-460, Thermo Fisher, USA). Crystalline forms in biochars were investigated using an X-ray diffraction analyser (XRD, D8, Bruker, Germany). The elemental of the biochars was analyzed by X-ray photoelectron spectroscopy (XPS, Escalab 250 xi, Thermo Fisher, USA). The degree of graphitization and disordered structural characteristics were revealed by Raman spectroscopy (Thermo Fisher, USA).

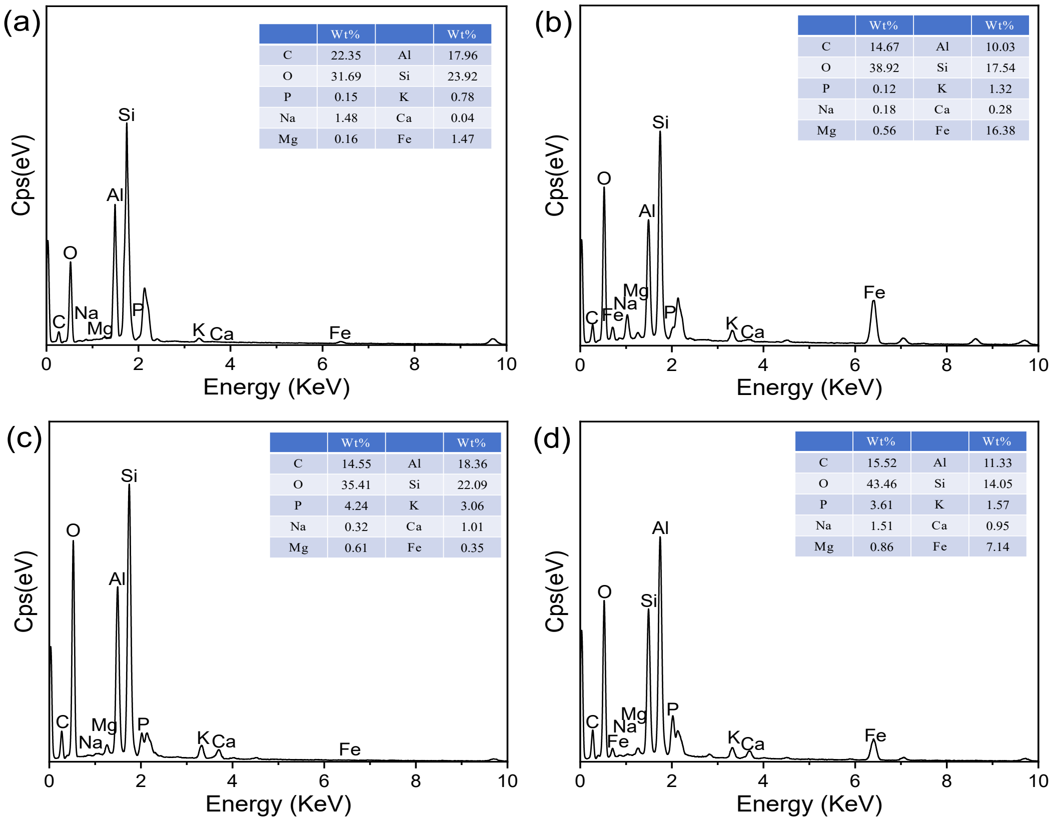

Fig. S1 EDS analysis of different adsorbents (a: SBC, b: Fe@SBC, c: P@SBC and d: P-Fe@SBC).

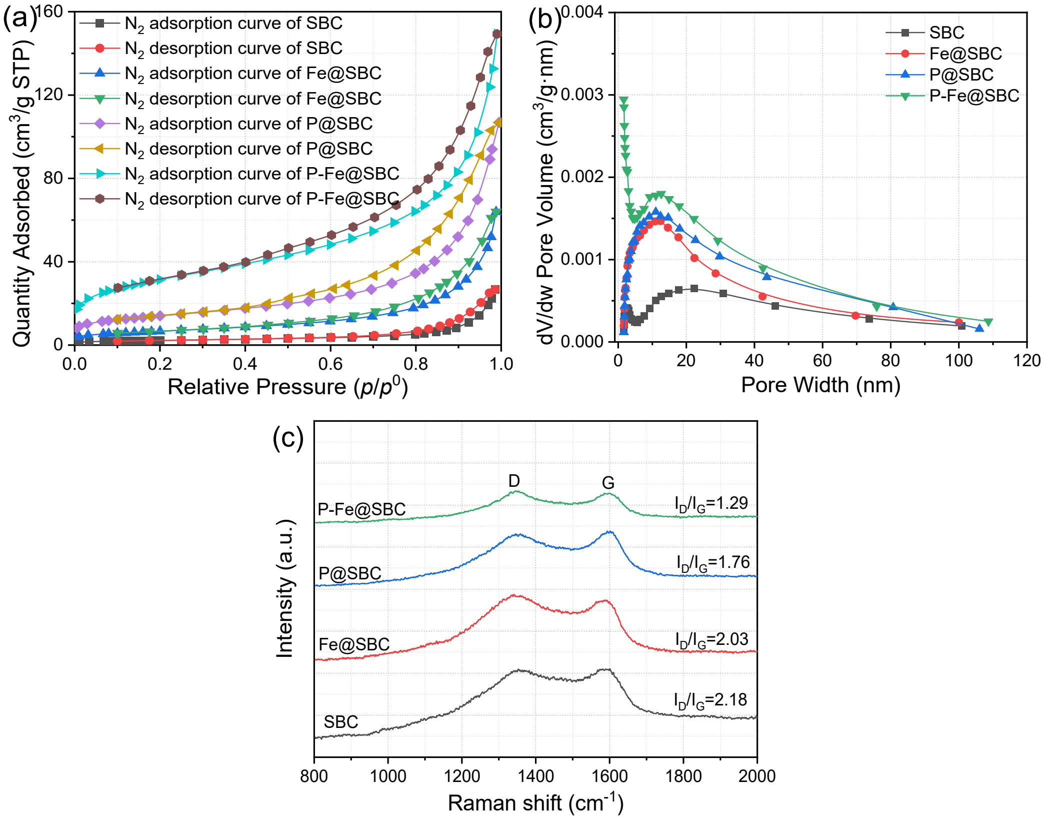

Fig. S2 N_2 adsorption-desorption isotherms (a), pore size distribution curves (b), Raman analysis (c) of different adsorbents.

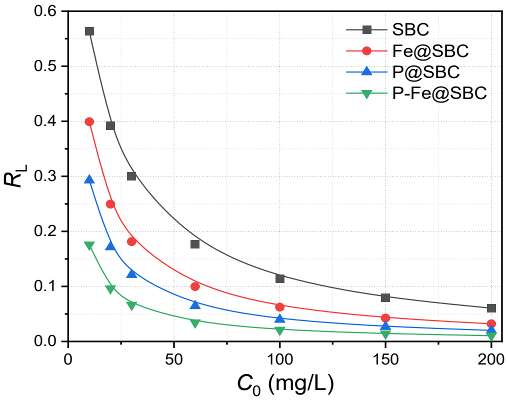

Fig. S3 Separation factors (R_L) of different adsorbents for Cd(II) removal

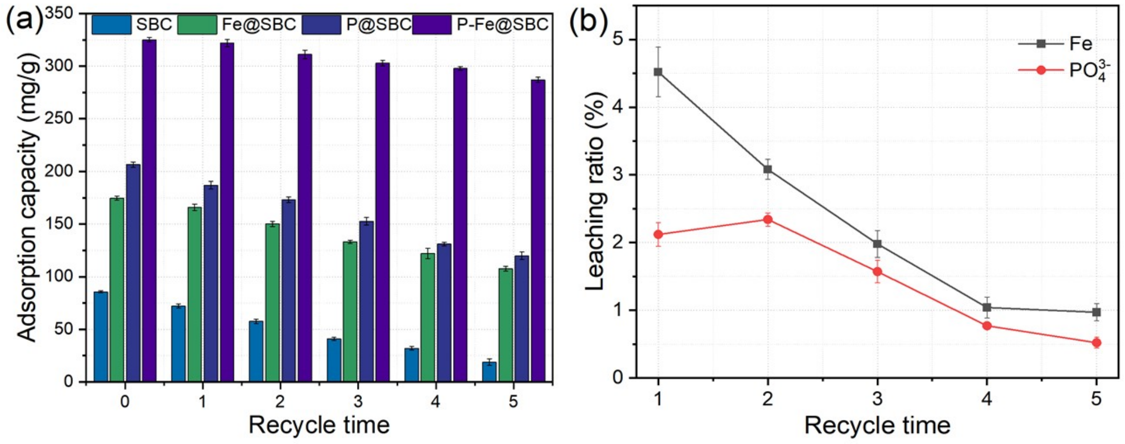

Fig. S4 Reusability of the adsorbents for Cd(II) removal over five adsorption-desorption cycles (a). Release concentration of Fe and phosphate after adsorption by different regenerated P-Fe@SBC (b).

Table S1 Comparison of the maximum adsorption capacity of different adsorbents for Cd^{2+}.

Adsorbent	Maximum adsorption capacity	References
Iron and silicon modified biochar	31.66	[1]
Chitosan@coconut shell-derived biochar	63.88	[2]
HCl-modified biochar	68.22	[3]
EDTA functionalized Mg/Al hydroxides modified biochar	204.53	[4]
Cysteine-grafted magnesium-modified biochar	223.7	[5]
Hydroxyl-functionalized Fe/Ni-biochar	229.52	[6]
Multifunctional magnetic biochar	292	[7]
P-Fe@SBC	326	This work

1. H. Wu, L. N. Liu, Y. G. Zhuo, R. M. Ellam, K. S. Yan, J. C. Liu and J. C. Tang, *Bioresour. Technol.*, 2024, **401**, 130745.
2. G. H. Mo, J. Xiao and X. Gao, *Biomass Convers. Bior.*, 2022, **13**, 16737–16752.
3. J. W. Wu, T. Wang, Y. S. Zhang and W. P. Pan, *Bioresour. Technol.*, 2019, **291**, 121859.
4. Y. F. Wang, J. E. Li, L. Xu, D. Wu, Q. N. Li, Y. H. Ai, W. Liu, D. N. Li, Y. T. Zhou, B. Y. Zhang, N. Guo, Y. Tao and Y. Zhang, *Separation and Purification Technology*, 2024, **335**, 126199.
5. A. Y. Li, C. H. Ye, Y. H. Jiang and H. Deng, *Bioresour. Technol.*, 2023, **386**, 129515.
6. H. Wang, Q. Chen, H. X. Xia, R. R. Liu and Y. H. Zhang, *Separation and Purification Technology*, 2024, **328**, 125074.
7. G. H. Mo, F. P. Li, J. N. Xiao, Z. G. Han and Z. M. Zhang, *Chemical Engineering Journal*, 2025, **526**, 171319.
